# Diagnostic and Therapeutic Management of Upper Extremity Deep Vein Thrombosis

**DOI:** 10.3390/jcm9072069

**Published:** 2020-07-01

**Authors:** Floris T. M. Bosch, Marcello Di Nisio, Harry R. Büller, Nick van Es

**Affiliations:** 1 Department of Internal Medicine, Tergooi Hospitals, 1213 XZ Hilversum, The Netherlands; 2 Department of Vascular Medicine, Amsterdam Cardiovascular Science, Amsterdam UMC, University of Amsterdam, 1105 AZ Amsterdam, The Netherlands; h.r.buller@amsterdamumc.nl (H.R.B.); n.vanes@amsterdamumc.nl (N.v.E.); 3 Department of Medicine and Ageing Sciences, Gabriele D′Annunzio University, Via dei Vestini 31, 66100 Chieti, Italy; mdinisio@unich.it

**Keywords:** upper extremity deep vein thrombosis, epidemiology, diagnosis, treatment

## Abstract

Upper extremity deep vein thrombosis (UEDVT) accounts for 5% of all deep vein thromboses (DVTs). UEDVT may be complicated by post thrombotic syndrome and pulmonary embolism, and early recognition and prompt start of anticoagulant treatment are key. Primary UEDVT, also known as Paget-von Schrötter syndrome, is associated with repeated or sudden physical activity of the upper arm and venous outflow obstruction due to anatomical variations. Secondary UEDVT is often associated with malignancy or use of intravenous devices, such as central venous catheters or pacemaker leads. Although the diagnosis and treatment of UEDVT have many similarities with DVT of the lower extremities, knowledge of specific aspects regarding UEDVT is important to guide optimal management. In this review, we will discuss the epidemiology, diagnosis, and treatment of UEDVT based on the current literature.

## 1. Introduction

Upper extremity deep vein thrombosis (UEDVT) was first recognized by the English pathologist and surgeon Sir James Paget in 1855, who described two cases of flebitis of the upper extremity in his clinical lectures and essays. Paget had already recognized the risk of pulmonary embolism (PE) in patients with certain variants of flebitis. However, since vitamin K antagonists were not introduced until the 1950s, the optimal treatment was a mystery. This is characterized in his clinical lectures by the treatment of one of his two patients with “a milk diet, six leeches every third night, and three grains of mercury with chalk every night and morning” [1]. The Austrian internist Leopold von Schrötter described the same disease in 1884, and in 1949, the English surgeon Hughes coined the term Paget-von Schrötter syndrome [2]. When the relation with a thoracic outlet problem was confirmed, the term “effort thrombosis” was introduced, due to its relation with activity [3,4].

Today, UEDVT is categorized in primary UEDVT (i.e., Paget-von Schrötter syndrome) and secondary UEDVT, which in the majority of cases is caused by a central venous catheter (CVC), a pacemaker lead, or cancer [5]. Whereas the diagnostic and therapeutic management of deep vein thrombosis (DVT) of the lower extremities has been well established over the years, less is known about UEDVT due to its lower incidence. Nonetheless, UEDVT is not to be neglected, since it accounts for approximately 5% of all DVTs [6,7]. A fast and accurate diagnosis followed by effective treatment of UEDVT is important, since patients can develop post-thrombotic syndrome (PTS) of the arm or PE [6,8]. In this review, we summarize contemporary literature on the diagnostic and therapeutic management of both primary and secondary UEDVT.

## 2. Epidemiology

Primary UEDVT is characterized by compression of the axillary or subclavian vein, which causes blood flow obstruction. The most important risk factors are strenuous muscular effort and anatomical abnormalities, including an enlarged costoclavicular ligament, which is located between the clavicle and the first rib [9]. Due to physical activity and subsequent hypertrophy of the surrounding muscles, the vein can be significantly narrowed and thrombosis can develop [10]. There is a higher prevalence of antiphospholipid antibodies, factor V Leiden, and prothrombin gene mutations in patients with primary UEDVT as compared with the general population [11]. Patients often present with oedema, pain, discoloration of the arm, paraesthesia, weakness, or cyanosis [12,13]. Due to the association of primary UEDVT with physical activity and congenital anatomical abnormalities, the median age at diagnosis is only 30 years [10]. Primary UEDVT is a rare condition, the incidence is estimated to be between one and two per 100,000 patients per year with an equal distribution between the sexes [5,10,14]. 

**Figure 1:**
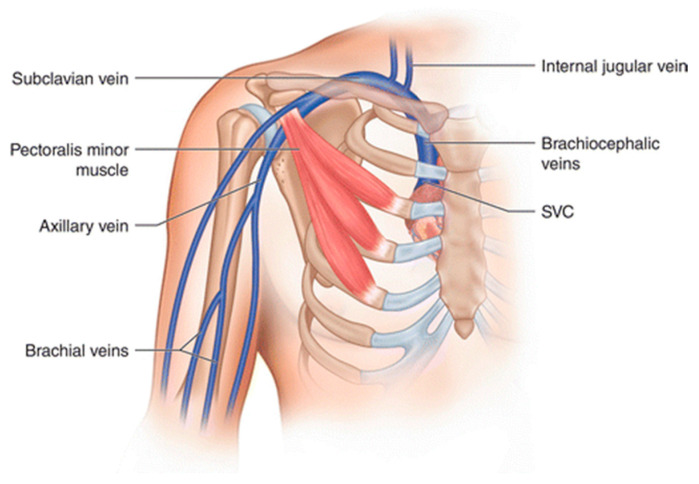
Anatomy of deep venous system of the upper extremity. Abbreviations: SVC, superior vena cava.

In secondary UEDVT, blood clots most frequently are located in the subclavian vein, but also in the jugular, axillary, brachial, or brachiocephalic veins. Rarely, more distal veins like the radial vein are involved (Figure 1) [15]. Patients usually have signs and symptoms similar to primary UEDVT, but can also report reduced mobility, signs of PE, or no symptoms at all [15]. The most important risk factors are malignancy (prevalence 22–64%) and indwelling lines (prevalence 10–93%) [5,15,16,17,18]. Additional risk factors are recent surgery or trauma, the use of hormone therapy, and hereditary or acquired thrombophilia [5,17,19]. Due to the strong association of secondary UEDVT with cancer and CVC, the mean age at presentation is about 60 years, which is substantially older than in primary UEDVT. The incidence of secondary UEDVT depends on the study population and follow-up duration. For example, the incidence of UEDVT was 1.8% in the PROTECT trial, which evaluated thromboprophylaxis in 3746 critically ill patients [20]. In the California Cancer Registry over a 10-year period, among 785,444 patients, 6088 (0.8%) developed UEDVT. The 24-month cumulative incidence differed across tumour types with the lowest incidence observed in prostate cancer (0.07%) and the highest in leukaemia (1.7%) [21]. Due to the increasing cancer incidence, improvements in survival, and widespread use of indwelling catheters, the incidence of UEDVT is expected to rise in parallel.

## 3. Diagnostic Management

### 3.1. Clinical Probability

For clinically suspected DVT of the lower extremities, the diagnostic pathway is well-established and based on validated clinical decision scores, such as the Wells and revised Geneva scores [22,23]. In patients with a low clinical probability, the diagnosis can be ruled out by a negative D-dimer, whereas those with a high clinical probability undergo ultrasonography. For patients with clinically suspected UEDVT, Constans and colleagues combined clinical signs and symptoms with risk factors for UEDVT to derive a clinical decision score comparable with those used for lower-extremity DVT [24]. This score was constructed in a derivation cohort of 140 patients with suspected UEDVT of whom 50 patients (36%) had the diagnosis objectively confirmed. Patients were assigned 1 point for either the presence of venous material (i.e., catheter in a subclavian or jugular vein or pacemaker), localized pain, or unilateral pitting oedema, while 1 point was subtracted when another diagnosis was considered at least as plausible as UEDVT. The sum score ranges from −1 to 3; patients with zero or less points were considered to be at low risk of UEDVT, those with a score of 1 at intermediate risk, and those with 2 or more points at high risk (Table 1). Then, the Constans score was internally validated in a cohort of 103 patients and externally validated in a cohort of 214 patients with clinically suspected UEDVT. In patients at low risk, the prevalence of UEDVT was 12%, 9%, and 13% in the derivation, internal validation, and external validation cohorts, respectively, as compared with 70%, 64%, and 69% in patients with high risk scores. The area under the curve for the three cohorts ranged from 0.68 in the derivation cohort to 0.78 in the external validation cohort. Taken together, these findings indicate that the diagnostic accuracy is not high enough to justify the use of the Constans rule as a standalone tool, it can, nonetheless, help clinicians to identify patients at high risk for whom imaging is warranted. 

### 3.2. D-Dimer Testing

The accuracy of D-dimer testing for diagnosing UEDVT was assessed in two studies. Merminod and colleagues prospectively included 52 patients with clinically suspected UEDVT; of whom 23 (44%) had active cancer, and 18 (35%) a permanent indwelling catheter. The sensitivity and specificity of D-dimer at the cut-off value of 500 µg/L were 100% (95% CI 78 to 100%) and 14% (95% CI 4 to 29%), respectively. The accuracy was consistent in the subgroups of patients with cancer or CVC [25]. In another prospective study by Sartori and colleagues, D-dimer testing was performed in 239 patients with suspected UEDVT of whom 39 patients (16.3%) had active cancer and 14 (5.9%) CVC. UEDVT was confirmed in 24 patients (10%) and superficial vein thrombosis in an additional 35 (14.6%). Using a cut-off of 500 µg/L, D-dimer testing had a sensitivity and specificity of 92% (95% CI 73 to 99%) and 60% (95% CI 52 to 67%), respectively [26]. Taken together, these two studies indicate that the sensitivity of D-dimer testing is as high as in patients with suspected lower-extremity DVT, making it an appropriate test for ruling out UEDVT in patients with a low clinical suspicion and normal D-dimer concentration. In patients with an elevated D-dimer level or patients with a high clinical suspicion, additional imaging remains necessary to confirm the diagnosis.

### 3.3. Imaging

Although venography is considered to be the gold standard for the diagnosis of UEDVT, it is not the first choice of imaging due to its invasive nature, radiation exposure, and risk of allergic contrast reactions [27]. Ultrasonography is often preferred as a less expensive and readily available alternative. In a systematic review, Di Nisio and colleagues summarized the evidence from nine studies that evaluated the diagnostic accuracy of ultrasonography in patients with suspected UEDVT using venography as the reference test. For compression ultrasonography, the pooled results showed a high sensitivity (97%, 95% CI 90 to 100%) and specificity (96%, 95% CI 87 to 100%). Doppler ultrasonography appeared to have a lower sensitivity (84%, 95% CI 72 to 97%) and comparable specificity (94%, 95% CI 86 to 100%), as did Doppler ultrasonography with compression (sensitivity 91%, 95% CI 85 to 97% and specificity 93%, 95% CI 80 to 100%) [28]. Surprisingly, Doppler ultrasonography combined with compression appeared to have a lower sensitivity than compression ultrasonography alone, despite the challenge to compress the subclavian vein due to its close anatomical relation with the clavicle. However, this finding comes from an indirect comparison and should be interpreted with caution because of the small sample size of many of the included studies resulting in wide confidence intervals, methodological limitations of the studies, and differences in patient populations that could have influenced the diagnostic accuracy. In a more recent prospective study of 483 patients with suspected UEDVT, the performance of serial ultrasonography was evaluated [29]. Whole-arm ultrasonography was normal in 319 patients (66%) and inconclusive in 21 (4.3%). In the latter group, ultrasound was repeated after five to seven days, and detected one patient (4.8%) with superficial vein thrombosis and two patients (9.5%) with DVTs. Of the 337 patients with a normal whole-arm ultrasonography, only one patient developed DVT during the three-month follow-up (failure rate 0.3%, 95% CI 0.1–1.7%). On the basis of these results, ultrasonography, with repeated imaging in case of inconclusive results, appears to safely exclude UEDVT, and therefore is widely accepted as the first-line imaging test in the diagnostic management of UEDVT.

When ultrasonography is inconclusive, computed tomography (CT) venography is often used to confirm or exclude UEDVT, despite the absence of data on its diagnostic accuracy. One small study assessed the degree of venous stenosis in 24 patients with thoracic outlet obstruction by comparing digital subtraction venography and CT venography. While CT was able to diagnose obstruction, only four patients in this study had concurrent thrombosis [30]. Magnetic resonance imaging (MRI) does not expose the patient to ionizing radiation and can be a useful option in the diagnosis of UEDVT. MRI was first studied by Baarslag and colleagues [31] who used two techniques, i.e., time-of-flight and gadolinium-enhanced three-dimensional (3D) MR venography. Of the 31 eligible patients with suspected UEDVT, MRI was not performed in 10 subjects due to various reasons (unable to lie flat, claustrophobia, too large for MR scanner, presence of osteosynthesis, or pacemaker). The overall sensitivity and specificity were poor for both MRI techniques, i.e., 71% and 89% for time-of-flight, and 50% and 80% for gadolinium 3D, respectively, as compared with contrast venography. A recent pilot study using non-contrast-enhanced magnetic resonance direct thrombus imaging showed promising results in three patients in whom UEDVT was already diagnosed with ultrasonography or venography. This technique, which can visualize the thrombus without the need for venous contrast, recently showed good performance in patients with suspected recurrent lower-extremity DVT [32,33]. A study with MRI direct thrombus imaging was recently completed in 63 subjects with suspected UEDVT, and results are eagerly awaited [34]. Although MRI could be a valuable method for the diagnosis of UEDVT, it is unlikely to become a first-line imaging test since it is costly, not widely available, time-consuming, and not suited for patients with claustrophobia or carrying internal devices such as pacemakers. It can be helpful, though, in patients in whom ultrasonography remains inconclusive after repeated measurements or when there is doubt about the diagnosis in patients who had a previous UEDVT.

### 3.4. Diagnostic Algorithm

**Figure 2:**
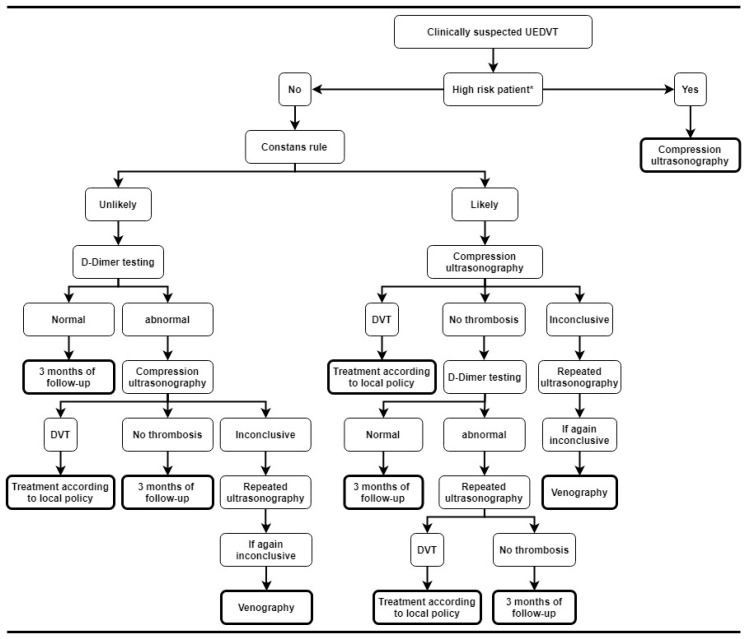
Diagnostic algorithm for upper extremity deep vein thrombosis based on the Armour study. * High risk patients: patients with malignancy, central venous catheter, or pacemaker, inpatients, or elderly (≥75 year old). Abbreviations: UEDVT, upper extremity deep vein thrombosis; DVT, deep vein thrombosis.

The feasibility and safety of a diagnostic algorithm for UEDVT combining a clinical decision rule, D-dimer testing, and ultrasound were evaluated in the ARMOUR study, which was a multicentre prospective study on 406 patients with clinically suspected UEDVT (Figure 2) [35]. Patients were first classified as “UEDVT likely” or “UEDVT unlikely” based on a dichotomized Constans score. Patients with a Constans score of ≤1 were classified as “UEDVT unlikely”. In this group, quantitative D-dimer testing was performed and if the level was below 500 µg/l, UEDVT was considered to be excluded. Patients with a Constans score ≥2 were classified as “UEDVT likely”. These patients and those with high D-dimer levels underwent compression ultrasonography. In patients with “UEDVT likely” and no evidence of thrombosis on ultrasonography, D-dimer levels were measured. If D-dimer was normal, UEDVT was excluded, otherwise a second ultrasonography was performed. On the basis of a low Constans score and normal D-dimer, imaging could be withheld in 87 patients (21%), of whom none developed VTE during a three-month follow-up. In the remaining 319 patients, superficial venous thrombosis and UEDVT were diagnosed by initial testing in 54 (13%) and 103 (25%) patients, respectively. Of 249 patients with a normal diagnostic work-up, one patient developed UEDVT during three months of follow-up, resulting in a failure rate of 0.4% (95% CI 0 to 2.2) which supports the safety of this diagnostic algorithm.

In a post hoc analysis of the ARMOUR study, in subgroups of patients at high risk of UEDVT, ultrasonography could be withheld in only 4% of patients with a central venous catheter and 5% of inpatients, which was significantly lower than the 21% in the overall population. This was also seen in cancer patients (11%) and patients 75 years or older (13%). One explanation the authors provided was that patients with cancer, high age, or indwelling lines could have had high D-dimer results regardless of an UEDVT, resulting in false positive results. Additionally, patients with central venous catheters already scored 1 point in the clinical decision rule, making it more difficult to be classified as ”UEDVT unlikely”, which resulted in more ultrasonography [36]. By applying an age-adjusted D-dimer threshold (i.e., a patient’s age multiplied by 10 µg/L in those older than 50 years), the number of patients for whom ultrasonography could be safely withheld was marginally increased from 21% to 25% [37]. Importantly, this approach has not yet been validated in management studies.

Because of the aforementioned findings, D-dimer testing and the Constans rule should not be used in patients with a CVC or pacemaker, inpatients, elderly (≥75 year), or patients with cancer. In these patients clinicians should proceed to compression ultrasonography directly (Figure 2). In all other patients, the dichotomized Constans rule can be applied followed by D-dimer testing in those “UEDVT unlikely” or immediate ultrasonography in patients classified as “UEDVT likely”. Repeated ultrasonography should be performed after three to five days in case of an inconclusive ultrasound or when ultrasonography shows no thrombosis but patients have a high subsequent D-dimer result (Figure 2) [38].

## 4. Treatment

Adequate treatment of UEDVT is important to prevent PE, DVT recurrence, and PTS of the arm. A recent analysis using the RIETE registry compared outcomes for UEDVT from different causes (*n* = 2272) and compared them with DVT of the lower extremities [39]. The incidence of recurrent PE in patients with provoking risk factors (i.e., recent immobility, surgery, bone fracture, active cancer, estrogen use, pregnancy, puerperium, or long-term travel) was 2.5 per 100 patient years (95% CI 1.16–4.74), which was similar to patients with DVT of the lower extremities (HR 1.14, 95% CI 0.53–2.20). In patients with no provoking risk factors, the incidence of recurrent PE was rare (0.24 per 100 patient years, 95% CI 0.01–1.17), and it occurred less frequently than in patients with DVT of the lower extremities, albeit not statistically significant (HR 0.28, 95% CI 0.01–1.39). Patients with catheter-related UEDVT had the highest risk of PE with 3.82 events per 100 patient years (95% CI 2.26–6.06). The recurrence rate in patients with UEDVT was also reported in a systematic review by Thiyagarajah and colleagues which included 3350 patients from 62 studies [8]. During a mean follow-up of six months, recurrence was more common in secondary UEDVT with a pooled incidence of 16% (95% CI 0.6–31) as compared with 6.4% (95% CI 4–9) in patients with primary UEDVT. Potential explanations for this difference could include the large proportion of patients with primary UEDVT who had undergone decompression therapy and the persistent status of hypercoagulability in patients with secondary UEDVT due to cancer or a CVC. In addition to recurrence, PTS is an important problem in patients with UEDVT. Methods to quantify PTS in UEDVT are often extrapolated from data in patients with DVT of the lower extremities. A modified Villalta score, which assesses the severity of PTS in DVT of the lower extremities, has been used in studies to assess the severity of PTS in the upper extremity [40,41]. This score uses five symptoms (pain, cramps, heaviness, pruritus, and paraesthesia) and six physical signs (edema, prominent veins on arm, prominent veins over shoulder or anterior chest wall, redness, tenderness, and dependent cyanosis) to assess the severity of PTS [40]. Patients with PTS of the upper extremity, according to this modified Villalta score, have significantly more functional disability and poorer quality of life [40]. Thiyagarajah and colleagues also reported on the risk of PTS in UEDVT. The authors found that the risk of PTS was higher in primary UEDVT with an incidence of 20% (95% CI 11–30) as compared with 14% in secondary UEDVT (95% CI 0–30) during the mean follow-up of six months (range three months to nine years) [8]. Although PTS is a common complication after UEDVT, no trials have been performed to assess the benefit of compression therapy. Future studies are needed to assess the benefit of compression sleeves, until then it is not routinely recommended in patients with UEDVT [42].

### 4.1. Anticoagulation

To date, no randomized controlled trials have been performed to evaluate anticoagulant treatment for UEDVT. There is only limited evidence available from cohort studies or extrapolated from studies in patients with lower limb DVT. There is general consensus that thrombosis of the axillary or more proximal veins should be treated with anticoagulation therapy for at least three months [43]. It is unclear whether patients with distal UEDVT benefit from anticoagulation and some authors have suggested using prophylactic doses or even no treatment with clinical surveillance [43]. Until about a decade ago, therapeutic options included either low-molecular-weight heparin (LMWH) or vitamin K antagonists (VKAs). These agents were compared only in one nonrandomized study in which 67 patients with cancer-related UEDVT received either dalteparin for five to seven days followed by warfarin or dalteparin alone for three months. In this study, 52 patients (78%) had an indwelling catheter. There were no recurrent DVT during the three-month follow-up in both groups, whereas one patient on warfarin experienced major bleeding [44]. Although both treatment regimens appeared to be effective and safe, firm conclusions could not be drawn given the small sample size and nonrandomized design. In the larger RIETE registry, 512 patients with UEDVT were enrolled, of whom 92 patients (18%) had cancer, 124 patients (24%) had a catheter, and 104 patients (20%) had both; 249 patients (50%) were treated with VKA and 244 patients (49%) received LMWH. During three months of follow-up, 11 patients (2.1%) experienced major bleeding, 12 patients (2.3%) developed recurrent DVT, and nine patients (1.8%) had PE. These findings are very similar to those observed in patients with DVT of the lower extremities [6]. Since outcomes were not provided for patients with LMWH and VKA separately, these data can unfortunately not be used to guide decisions about the type of anticoagulant treatment. 

There is some doubt whether DOACs, including the factor Xa-inhibitors rivaroxaban, apixaban, and edoxaban, as well as the thrombin inhibitor dabigatran, effectively prevent thrombosis related to a foreign body, such as CVCs, as they may not adequately suppress coagulation driven by the intrinsic pathway. For example, there was an increased risk of thromboembolic and bleeding complications in patients with a mechanical heart valve treated with dabigatran in the RE-ALIGN study [45]. Data on direct oral anticoagulants (DOACs) for the treatment of UEDVT are very scarce, and include only small studies that mainly evaluated rivaroxaban (Table 2) [17,46,47,48,49,50]. Nonetheless, since DOACs are recommended for patients with DVT of the lower extremities, these agents are increasingly being prescribed in patients with UEDVT [51,52,53]. The rates of recurrent VTE and major bleeding with DOACs ranged from 0 to 3.6% and from 0 to 10%, respectively. Fan and colleagues performed a prospective cohort study in which patients with cancer and CVC-related UEDVT were treated with rivaroxaban 20 mg once daily or LMWH followed by warfarin for a total of three months. There were no patients who developed recurrent thrombosis or major bleeding [46]. In a more recent retrospective, single-center study, Houghton and colleagues compared DOACs (apixaban or rivaroxaban) to LMWH or warfarin. Overall, 125 patients (60%) had malignancy and 107 patients (51%) catheter-associated UEDVT. During the three-month follow-up, patients treated with DOACs had one (1%) recurrent thrombosis and no major bleeding. Two patients (2%) developed clinically relevant non-major bleeding. In patients treated with LMWH or warfarin, there was one patient (0.9%) with recurrent thrombosis, three patients (2.8%) with major bleeding, and one patient (0.9%) with clinically relevant non-major bleeding. It is unclear how many patients were treated with apixaban or rivaroxaban [50]. Four studies evaluated rivaroxaban for UEDVT. In a prospective cohort on cancer patients with CVC-related UEDVT, a substantial proportion developed major bleeding (10%) during the anticoagulant therapy. This could highlight the high baseline bleeding risk in the oncology population. Additionally, one recurrent VTE, presenting as fatal PE, occurred during the three month follow-up [47]. In the other three studies evaluating rivaroxaban, it appears that the risk of recurrence and bleeding were similar to those observed in the RIETE registry. 

Taken together, current evidence suggests that anticoagulation with either LMWH, VKA, or DOAC is effective and safe. In cancer patients with lower limb DVT, VKAs have a lower efficacy and similar safety as compared with LMWH [54]. Therefore, current guidelines recommend against VKA for the treatment of cancer-associated thrombosis [55,56]. Current guidelines do recommend LMWH, edoxaban, and rivaroxaban as treatment options for patients with cancer. The recently published CARAVAGGIO and ADAM VTE trials, which randomized patients with cancer and VTE to either apixaban or dalteparin, demonstrated that apixaban is also an effective and safe treatment option [57,58]. In the ADAM VTE trial, 15.3% of included patients had an UEDVT as the qualifying event. Out of two major bleeds in the dalteparin group, one bleeding event (0.7%) was observed in a patient with an UEDVT as the qualifying event. There were no additional subgroup analyses performed in patients with UEDVT only. Although evidence from direct comparison is missing for UEDVT, LMWH or DOACs could be preferable over VKAs for the treatment of cancer-associated UEDVT.

Two ongoing studies are currently evaluating the efficacy and safety of apixaban for the treatment of UEDVT (NCT03100071 and NCT02945280) [59]. The Catheter-3 study aims to include 70 patients with cancer and CVC-related UEDVT, who will receive LMWH for one week followed by apixaban 5 mg twice daily for 11 weeks. Outcomes will be line failure, VTE recurrence, and bleeding. The ARM-DVT will include 375 patients, who will be treated with apixaban 10 mg twice daily for one week followed by apixaban 5 mg twice daily for 11 weeks. The primary efficacy outcome will be symptomatic VTE and VTE-related death. The safety outcome consists of major and clinically relevant non-major bleeding. Both studies are expected to complete recruitment this year. 

### 4.2. Central Venous Catheter Management

In patients with CVC-related UEDVT, anticoagulation therapy should be started regardless of catheter removal [43]. The catheter should be removed when it is no longer needed or functional, followed by anticoagulation therapy for at least three months.

This strategy was studied in a single-centre cohort study that included 74 patients with CVC-related UEDVT, who were treated with dalteparin for five days followed by warfarin for a total of three months without catheter removal. When a catheter lumen obstruction developed, patients received a maximum of 2 mg of tissue plasminogen activator (tPA) up to two times per blocked lumen to attempt to unblock the catheter. At the end of the three-month follow-up, 42 (57%) patients still had the CVC in place and no recurrent VTE was observed. There were three (4.7%) episodes of major bleeding, including one fatal upper gastrointestinal bleed [60].

### 4.3. Thrombolysis

Thrombolysis is suggested as a potential treatment option for acute UEDVT of the axillary of more proximal veins. There are no RCTs on the use of thrombolysis in patients with UEDVT [61]. Three observational studies compared thrombolytic therapy and anticoagulation with anticoagulation alone in patients with primary and secondary UEDVT. Two of these studies, totalling 39 patients, demonstrated improved recanalization and symptom resolution with thrombolytic agents [62,63]. In another retrospective cohort study that included 92 patients, the higher recanalization obtained with thrombolysis was offset by a rate of bleeding as high as 21%. Furthermore, there was no difference in PTS during a median follow-up of 40 months [64]. On the basis of these and several single-arm studies, the American College of Chest Physician (ACCP) guidelines have suggested oral anticoagulation alone over thrombolysis [53]. However, thrombolysis can be considered in highly selected patients who have severe symptoms, thrombus involving the subclavian and axillary vein, symptoms for less than 14 days, good functional status, life expectancy of more than one year, and a low risk of bleeding [43]. Thrombolysis is not advised in patients presenting themselves more than 14 days after the onset of symptoms, as the risk of bleeding increases by each day of delay from the beginning of symptoms [65]. To decrease the risk of bleeding, catheter-directed thrombolysis (CDT) is advised over systemic thrombolysis. Following thrombolysis, anticoagulation should be started and continued for a minimum of three months to minimize the risk of recurrence. Since the vast majority of patients with secondary UEDVT have a poor functional status, high bleeding risk, or asymptomatic VTE, in clinical practice, these criteria are likely only met by patients with primary UEDVT.

### 4.4. Thrombolysis Followed by Surgical Decompression in Primary Upper Extremity Deep Vein Thrombosis (UEDVT)

Although not mentioned in the ACCP guidelines, many centers apply a treatment regimen of thrombolysis followed by surgical decompression in the case of primary UEDVT with more acute and severe symptoms [66,67,68]. This surgical procedure consists of removal of the first rib and the costoclavicular ligament with the goal to relieve symptoms by restoring normal bloodflow in the subclavian vein [10,66]. A meta-analysis of twelve series including a total of 684 patients found that patients who were treated with first rib resection after thrombolysis, as compared with thrombolysis alone, were significantly more likely to have symptom relief at last follow-up (95% vs. 54%, *p* < 0.0001), as well as vein patency (98% vs. 48%, *p* < 0.0001) [69,70]. There is debate about the timing of surgical decompression. A recent systematic review compared data from six case series with a total of 126 patients. In this meta-analysis, patients were divided into the following two groups: early surgical intervention (<2 weeks after thrombolysis) and postponed intervention (>2 weeks after thrombolysis). At the end of follow-up, 89% in the early intervention group versus 90% in the postponed intervention group had minimal or no symptoms. During follow-up, 18% of the early group had a recurrent event as compared with 31% in the postponed group. Therefore, the authors concluded that early intervention after thrombolysis seemed to be as safe and effective as postponed intervention [67]. Due to the lack of randomized trials and mostly data from small retrospective studies, no surgical guidelines currently propose a strict treatment regimen for primary UEDVT. However, based on several series, CTD combined with surgical decompression seems to be a safe and effective treatment regimen in patients who meet the criteria for thrombolysis according to the ACCP guidelines [43].

## 5. Conclusions

UEDVT requires early recognition and treatment to prevent complications, such as recurrence, PTS, and pulmonary embolism. A diagnostic strategy based on the sequential assessment of clinical factors and D-dimer testing can avoid imaging in about a quarter of patients. When imaging is required, ultrasound is widely used as the first-line imaging test and if inconclusive, it can be followed by a second ultrasonography, CT venography, or MRI. Patients with confirmed UEDVT should be treated with anticoagulation therapy for a minimum of three months. Longer treatment can be considered in patients with active cancer or CVC-related UEDVT until the catheter is removed. The preferred treatment options are DOACs, VKA, or LMWH. Thrombolysis can be considered in patients with severe symptoms and low risk of bleeding, such as younger patients with primary UEDVT. In the case of outside compression on the axillosubclavian vein, first rib resection can be considered.

## Figures and Tables

**Table 1:** Constans score.

Symptoms	Points
Venous material including catheter in a subclavian or jugular vein or pacemaker	+1
Localized pain	+1
Unilateral pitting edema	+1
Other diagnosis at least as plausible	−1
**Risk for UEDVT**	**Total Score**
Low risk	≤0
Intermediate risk	1
High risk	≥2

UEDVT, upper extremity deep vein thrombosis.

**Table 2:** Direct oral anticoagulants for the treatment of upper extremity deep vein thrombosis.

Author	Study Design	Patients	Duration	Treatment	Patient Characteristics	Outcomes
Laube (2017) [48]	Retrospective cohort	*n* = 83	3 months	Rivaroxaban (100%)	Age: 62 Male 28% CVC: 83 (100%) Malignancy: 100%	Recurrence: 3 (3.6%) CNRMB: 1 (1.2%) MB: 2 (2.4%)
				No control	-	-
Fan (2017) [46]	Prospective cohort	*n* = 84	3 months	Rivaroxaban *n* = 44 (52%)	Age: 50 Male: 57% CVC: 100% Malignancy: 100%	Recurrence: 0 CRNMB: 7.3% MB: 0
				LMWH/Warfarin *n* = 40 (48%)	Age: 51 Male: 56% CVC: 100% Malignancy: 100%	Recurrence: 0 CRNMB: 11.4% MB: 0
Montiel (2017) [17]	Retrospective cohort	*n* = 55	3–6 months	Rivaroxaban (84%) Apixaban (13%) Dabigatran (4%)	Age: 49 Male: 49% CVC: 20 (36%) Malignancy: 10 (18%)	Recurrence: 1 (2%) CRNMB: 1 (2%) MB: 0
				No control	-	-
Davies (2018) [47]	Prospective cohort	*n* = 70	3 months	Rivaroxaban (100%)	Age: 54.1 Male: 33% CVC: 100% Malignancy: 100%	Recurrence: 1 (1.43%) CRNMB: 4 (5.7%) MB: 7 (10%)
				No control	-	-
Schastlivtsev (2019) [49]	Prospective cohort	*n* = 30	6 months	Rivaroxaban (100%)	Age: 52 Male: 43% CVC: 4 (13.3%) Malignancy: 2 (6.7%)	Recurrence: 0 CRNMB: 2 (6.7%) MB: 0
				No control	-	-
Houghton (2020) [50]	Retrospective cohort	*n* = 210	3 months	Apixaban/Rivaroxaban *n* = 102 (49%)	Age: 57 Male: 55% CVC: 45 (44%) Malignancy: 53 (52%)	Recurrence: 0 CRNMB: 5 (6.8%) MB: 0
				LMWH/Warfarin *n* = 108 (51%)	Age: 62 Male: 63% CVC: 62 (57%) Malignancy: 72 (67%)	Recurrence: 1 (1.6%) CRNMB: 1 (1.6%) MB: 2 (3.3%)

CVC, central venous catheter; CRNMB, clinically relevant non-major bleeding; MB, major bleeding.
